# Exploring the Potential of Claude 3 Opus in Renal Pathological Diagnosis: Performance Evaluation

**DOI:** 10.2196/65033

**Published:** 2024-11-15

**Authors:** Xingyuan Li, Ke Liu, Yanlin Lang, Zhonglin Chai, Fang Liu

**Affiliations:** 1 Department of Nephrology West China Hospital Sichuan University Chengdu China; 2 Department of Diabetes Central Clinical School Monash University Melbourne Australia

**Keywords:** artificial intelligence, Claude 3 Opus, renal pathology, diagnostic performance, large language model, LLM, performance evaluation, medical diagnosis, AI language model, diagnosis, pathology images, pathologist, clinical relevance, accuracy, language fluency, pathological diagnosis

## Abstract

**Background:**

Artificial intelligence (AI) has shown great promise in assisting medical diagnosis, but its application in renal pathology remains limited.

**Objective:**

We evaluated the performance of an advanced AI language model, Claude 3 Opus (Anthropic), in generating diagnostic descriptions for renal pathological images.

**Methods:**

We carefully curated a dataset of 100 representative renal pathological images from the *Diagnostic Atlas of Renal Pathology* (3rd edition). The image selection aimed to cover a wide spectrum of common renal diseases, ensuring a balanced and comprehensive dataset. Claude 3 Opus generated diagnostic descriptions for each image, which were scored by 2 pathologists on clinical relevance, accuracy, fluency, completeness, and overall value.

**Results:**

Claude 3 Opus achieved a high mean score in language fluency (3.86) but lower scores in clinical relevance (1.75), accuracy (1.55), completeness (2.01), and overall value (1.75). Performance varied across disease types. Interrater agreement was substantial for relevance (κ=0.627) and overall value (κ=0.589) and moderate for accuracy (κ=0.485) and completeness (κ=0.458).

**Conclusions:**

Claude 3 Opus shows potential in generating fluent renal pathology descriptions but needs improvement in accuracy and clinical value. The AI’s performance varied across disease types. Addressing the limitations of single-source data and incorporating comparative analyses with other AI approaches are essential steps for future research. Further optimization and validation are needed for clinical applications.

## Introduction

Artificial intelligence (AI) has demonstrated remarkable capabilities in analyzing complex medical data and assisting clinical decision-making across various fields [1]. In particular, AI’s potential for interpreting histopathological images has been increasingly recognized, offering novel insights into disease pathogenesis and diagnosis [2]. However, the application of AI in renal pathology, a field characterized by high complexity and variability, remains relatively unexplored. Recent advancements in natural language processing, such as the development of large language models (LLMs) like GPT-3 (OpenAI) and Claude 3 Opus (Anthropic), have opened up new possibilities for AI-assisted pathological diagnosis [3,4]. These models can capture semantic and contextual information from textual data and generate coherent, human-like responses. Despite the proven utility of AI in other pathology domains, such as oncology and dermatology, its performance and feasibility in renal pathology have not been systematically evaluated. To address this gap, we conducted a pioneering study to investigate the potential of using Claude 3 Opus, a state-of-the-art AI language model, for renal pathological diagnosis. By assessing the model’s ability to generate accurate and clinically relevant diagnostic descriptions for a wide range of renal pathologies, we aimed to provide initial evidence and insights into the strengths and limitations of AI in this challenging field.

## Methods

We carefully curated a dataset of 100 representative renal pathological images from the *Diagnostic Atlas of Renal Pathology* (3rd edition) [5]. The image selection process aimed to cover a wide spectrum of common renal diseases, ensuring a balanced and comprehensive dataset. The number of images per disease type ranged from 2 to 9 (eg, immunoglobulin A nephropathy: n=5; acute tubular injury: n=4; diabetic nephropathy: n=9), proportional to the relative prevalence and morphological diversity of each condition. Multimedia Appendix 1 provides the complete list of images and their corresponding disease labels.

**Figure 1:**
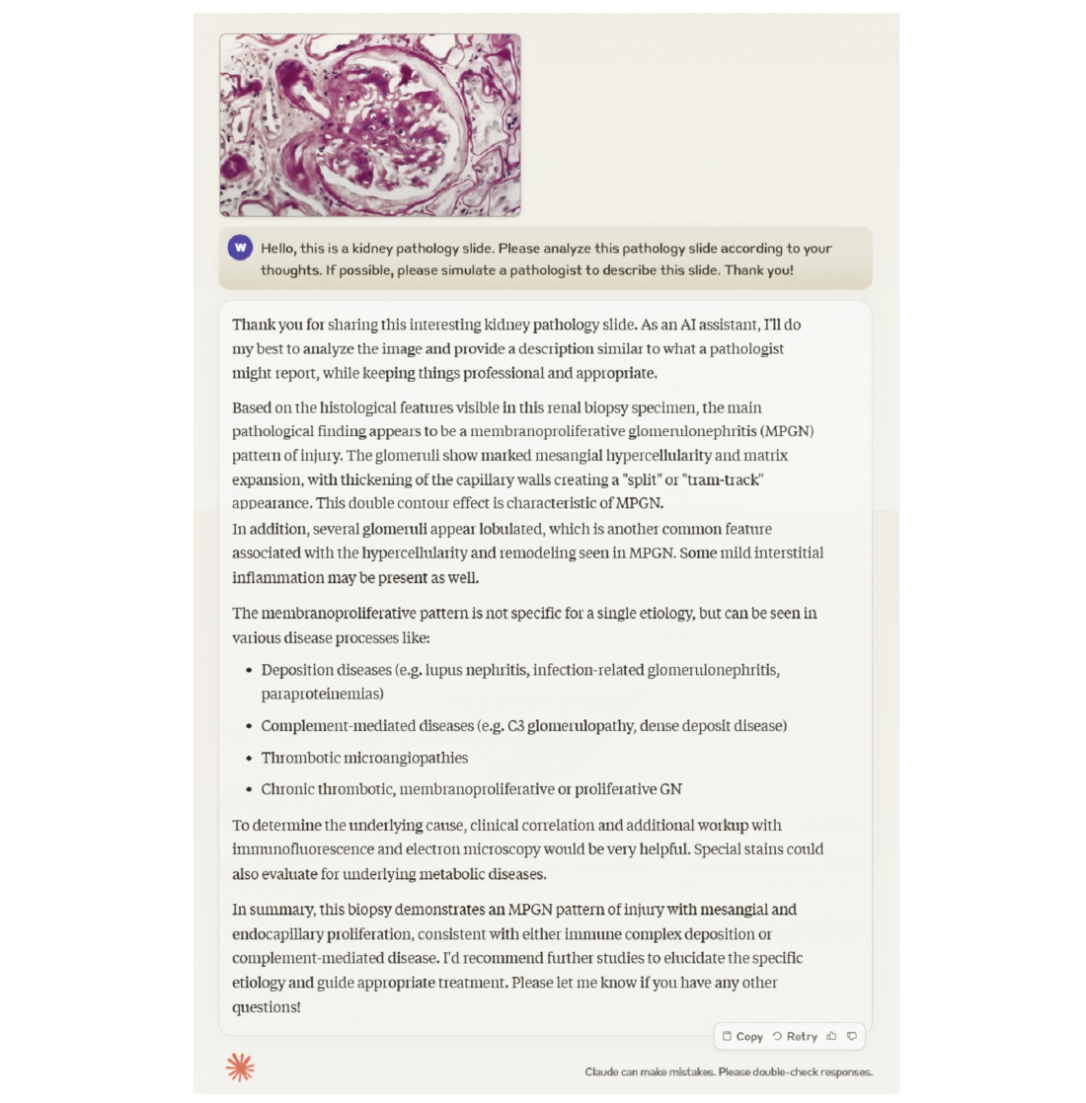
Example of a prompt to Claude 3 Opus and its response.

Claude 3 Opus, an advanced AI language model, was used to generate diagnostic descriptions for each image. For each image, the model was given the following prompt: “Describe the key morphological features and provide a diagnostic impression for this renal biopsy image.” No additional disease-specific information or background knowledge was provided, allowing us to assess Claude 3 Opus’s standalone performance in renal pathology interpretation. The generated descriptions were evaluated by 2 experienced renal pathologists using a comprehensive 5-point scale across 5 key dimensions: clinical relevance, accuracy, language fluency, detail completeness, and overall clinical value. These evaluation dimensions were selected based on established frameworks for assessing the quality and utility of pathology reports [6,7]. They collectively cover the essential aspects of an effective pathology description, from clinical applicability and correctness to clarity and comprehensiveness. Descriptive statistics (means and SDs) of the evaluation scores were calculated for each pathological type. The interrater agreement between the 2 pathologists’ scores was assessed using the Cohen κ statistic. Figure 1 shows an example of a prompt to Claude 3 Opus and its response.

## Results

The performance evaluation results for Claude 3 Opus in generating renal pathological descriptions are presented in Table 1. The AI model achieved a high overall score in language fluency (mean score 3.86, SD 0.68), indicating its ability to produce grammatically correct and easily readable reports. However, the model’s performance in other key aspects was suboptimal, with lower scores for clinical relevance (mean score 1.75, SD 0.77), accuracy (mean score 1.55, SD 0.66), detail completeness (mean score 2.01, SD 0.84), and overall clinical value (mean score 1.75, SD 0.74). Notably, the AI model’s performance varied across different renal pathological types. Higher scores (>4) were observed for certain diseases, such as membranoproliferative glomerulonephritis and subacute bacterial endocarditis–associated glomerulonephritis, suggesting the model’s potential in assisting the diagnosis of these specific conditions. Conversely, the model’s performance was subpar for several other types, including acute interstitial nephritis (mean score 1.00, SD 0.00) and collapsing glomerulopathy (mean score 1.17, SD 0.24), indicating limitations in capturing their key diagnostic features. The inter-rater agreement analysis revealed substantial agreement between the 2 pathologists on clinical relevance (κ=0.627) and overall clinical value (κ=0.589), as well as moderate agreement on accuracy (κ=0.485) and detail completeness (κ=0.458).

**Table 1:** Performance of Claude 3 Opus in generating descriptions for 100 renal pathological images across 27 disease types.

Standard pathological diagnosis	Clinical relevance, mean score	Accuracy of description, mean score	Language fluency, mean score	Detail completeness, mean score	Overall clinical applicability, mean score
Immunoglobin A nephropathy (n=5)	2.10	1.10	4.30	2.90	1.90
Immunoglobin G4–related tubulointerstitial nephritis (n=3)	1.67	1.17	4.33	2.50	1.67
Proliferative glomerulonephritis with monoclonal deposits (n=3)	2.33	1.83	4.17	2.83	2.50
Autosomal dominant polycystic kidney disease (n=2)	1.25	1.50	3.75	2.50	1.75
Henoch-Schönlein purpura nephritis (n=5)	1.10	1.10	3.90	2.00	1.40
Acute postinfectious glomerulonephritis (n=4)	1.63	1.38	3.88	2.38	1.63
Acute interstitial nephritis (n=3)	1.17	1.00	3.67	1.50	1.00
Acute tubular injury (n=4)	1.25	1.38	3.50	1.88	1.38
Acute pyelonephritis (n=4)	1.38	1.38	3.88	2.38	1.63
Focal segmental glomerulosclerosis (n=4)	1.63	1.75	3.75	2.25	1.88
Anti–glomerular basement membrane antibody–mediated glomerulonephritis (n=4)	2.38	2.38	4.00	2.75	2.63
Chronic interstitial fibrosis and tubular atrophy (n=2)	1.50	1.00	4.00	2.00	2.00
Chronic pyelonephritis (n=2)	2.00	1.50	4.00	2.00	2.00
Diffuse mesangial sclerosis (n=3)	1.83	1.50	3.50	1.50	1.83
Membranoproliferative glomerulonephritis (n=3)	4.17	4.00	3.83	3.67	4.00
Arterionephrosclerosis (n=4)	1.13	1.00	4.00	1.00	1.13
Medullary cystic disease (n=3)	1.33	1.17	3.83	1.67	1.33
Collapsing glomerulopathy (n=3)	1.17	1.17	3.50	1.17	1.17
Diabetic nephropathy (n=9)	1.50	1.44	3.61	1.50	1.44
Preeclampsia (n=8)	1.44	1.56	3.75	1.94	1.69
Fibrillary glomerulonephritis (n=2)	1.50	1.25	3.75	1.50	1.25
Microscopic polyangiitis (n=3)	1.83	1.67	3.83	1.67	1.83
Hemoglobinuric acute renal failure (n=2)	2.25	1.75	4.00	2.75	2.25
Thrombotic microangiopathy (n=3)	1.50	1.50	4.00	1.67	1.67
Subacute bacterial endocarditis–associated glomerulonephritis (n=3)	3.17	3.00	4.00	2.67	2.83
Scleroderma (n=4)	2.00	1.88	4.00	1.75	1.88
Kidney biopsies from healthy people (n=5)	1.50	1.10	3.50	1.40	1.30
Overall	1.75	1.55	3.86	2.01	1.75

## Discussion

### Principal Findings

This study provides initial evidence for the potential of advanced AI language models, such as Claude 3 Opus, in assisting renal pathological diagnosis. The model demonstrated promise in generating fluent and readable pathological descriptions, which could streamline the reporting process and alleviate pathologists’ workloads. However, the suboptimal performance in accuracy, clinical relevance, and overall value highlights the need for further improvement before clinical implementation. The interrater agreement analysis revealed substantial agreement for clinical relevance and overall value, but only moderate agreement for accuracy and completeness. This discrepancy might stem from the inherent subjectivity in evaluating granular aspects of pathology descriptions. Pathologists’ individual expertise, expectations, and interpretive styles could influence their assessments of accuracy and completeness. Developing standardized scoring rubrics and involving larger, multicenter expert panels in future studies could help mitigate this variability and improve evaluation reliability [8,9].

The AI model’s performance varied notably across different renal pathological types. The higher scores for conditions like membranoproliferative glomerulonephritis and infection-related glomerulonephritis could be attributed to their distinct morphological features, such as characteristic immune-complex deposits or structural alterations [10]. These overt patterns might be more readily discernible by the AI algorithms. Conversely, the lower performance for diseases like acute interstitial nephritis and collapsing glomerulopathy might reflect their subtler or more heterogeneous histological manifestations [11], posing challenges for automated interpretation.

While Claude 3 Opus exhibited potential in generating fluent descriptions, the limited accuracy and clinical relevance underscore the challenges of applying LLMs to complex medical image data. As highlighted by recent studies [12,13], LLMs excel at processing textual information but may struggle with the intricacies of specialized visual tasks like histopathology interpretation. Continued research on architectures and training strategies tailored for medical vision applications is crucial for realizing the full potential of AI in this domain.

Our study’s reliance on images from a single atlas dataset may have introduced some biases and limits the generalizability to real-world clinical scenarios. Although the *Diagnostic Atlas of Renal Pathology* is widely recognized as a high-quality reference, external validation using diverse, multicenter biopsy datasets is essential to assess the robustness and transferability of our findings [14]. Future studies should prioritize prospective validation on independent clinical cohorts to establish the real-world performance of AI models like Claude 3 Opus.

Comparing the performance of Claude 3 Opus with other AI models or traditional diagnostic methods could offer valuable insights into its relative strengths and areas for improvement. While direct comparisons were beyond the scope of this initial study, recent work has reported promising results with deep learning–based approaches for renal pathology classification. Shen et al [15] developed a convolutional neural network (CNN) model that achieved an accuracy of 87.5% in classifying 6 common glomerular diseases. Similarly, Hermsen et al [16] demonstrated the effectiveness of a multiclass CNN for diagnosing various renal pathologies, with an area under the receiver operating characteristic curve ranging from 0.88 to 0.99. These studies highlight the potential of specialized AI architectures for renal pathology diagnosis, serving as benchmarks for evaluating the performance of LLMs like Claude 3 Opus. Future research should aim to conduct head-to-head comparisons and explore synergistic integrations of LLMs with image-based AI models to leverage their complementary strengths.

To fully realize AI’s potential in renal pathology, future research should focus on optimizing the model’s training process with comprehensive and balanced datasets, incorporating expert feedback into the learning process, and integrating AI with advanced digital pathology tools for more accurate and objective diagnoses. The scarcity of large-scale annotated datasets and concerns about AI interpretability remain major challenges to be addressed. Amidst these challenges, the collaboration between AI researchers, pathologists, and clinicians is crucial for developing reliable and clinically applicable AI models. Standardized evaluation frameworks and best practices for the responsible use of AI in pathology are also needed. As AI continues to advance, we anticipate its increasing role in enhancing diagnostic accuracy and efficiency, ultimately benefiting patient care.

### Conclusion

In conclusion, our study provides an initial assessment of Claude 3 Opus’s potential for AI-assisted renal pathology diagnosis. While the model showed promise in generating fluent descriptions, improvements in accuracy and clinical relevance are necessary for practical implementation. The observed performance variations across disease types highlight the need for targeted model optimizations. Addressing the limitations of single-source data and incorporating comparative analyses with other AI approaches are essential steps for future research. As AI continues to advance, close collaboration between pathologists, AI researchers, and clinicians will be instrumental in developing reliable, integrated diagnostic solutions that enhance patient care in renal pathology.

## Appendix

Multimedia Appendix 1 Complete list of images and their corresponding disease labels.

## Abbreviations

AI: artificial intelligence
CNN: convolutional neural network
LLM: large language model

## Data Availability

The data sets generated during and/or analyzed during this study are available from the corresponding author on reasonable request.
