# Correction: Exploring the Potential of Claude 3 Opus in Renal Pathological Diagnosis: Performance Evaluation

**DOI:** 10.2196/88845

**Published:** 2026-02-23

**Authors:** JMIR Publications Editorial Office

Following the publication of “Exploring the Potential of Claude 3 Opus in Renal Pathological Diagnosis: Performance Evaluation” [1], concerns were raised regarding a potential conflict of interest between reviewer X Liu and members of the author list. After review, it was determined that the authors and the reviewer were both affiliated with Sichuan University West China Hospital. This conflict of interest was not disclosed to the editorial office as per journal policy.

This article has been subsequently reassessed by the *JMIR Medical Informatics* editor in chief, who determined that the content of this review did not unduly influence the decision to accept the article. The decision to accept the submission was based primarily on the remaining reviewers’ comments and the previous editor in chief’s contemporary appraisal of the manuscript.

As such, the JMIR Publications Editorial Office issues this corrigendum to update the Conflicts of Interest statement as follows:

Reviewer X Liu was found to have undisclosed conflicts of interest with members of the author list under the terms of JMIR Publications policy. The editor in chief determined that the content of the reviews did not unduly influence the decision to accept the article. The decision to accept the submission was based primarily on the remaining reviewers’ comments and the editor in chief’s contemporary appraisal of the manuscript.

We regret that these issues were not identified before publication.

The correction will appear in the online version of the paper on the JMIR Publications website together with the publication of this correction notice. Because this was made after submission to PubMed, PubMed Central, and other full-text repositories, the corrected article has also been resubmitted to those repositories.
