# Supplementary material for: Exploring the Potential of Claude 3 Opus in Renal Pathological Diagnosis: Performance Evaluation
Source: JMIR Med Inform. 2024 Nov 15;12:e65033. doi: 10.2196/65033 (PMC11607560; doi:10.2196/65033)
Supplement: Multimedia Appendix 1 [file medinform_v12i1e65033_app1.pdf]

Number	Standard pathological diagnosis	Source	Pathological diagnosis
1	IgA nephropathy	Standard pathological description	IgA nephropathy. There is mild mesangial matrix expansion and a mild increase in mesangial cellularity, with three or more nuclei in most mesangial areas in this early case of iga nephropathy (periodic acid－schiff, ×200).
		Claude	This renal biopsy section shows diffuse proliferative glomerulonephritis. There is marked global hypercellularity of the glomerular tufts with numerous proliferating endothelial and mesangial cells nearly obliterating the capillary lumens. The proliferative changes appear active and diffuse, involving nearly all glomeruli in the section. Some wireloop lesions are evident, suggestive of lupus nephritis class IV. The tubules, interstitium and blood vessels are unremarkable in this field. Overall, the findings are consistent with diffuse proliferative lupus nephritis, ISN/RPS class IV.
2		Standard pathological description	IgA nephropathy. There is marked mesangial expansion, with segmental hypercellularity extending out to peripheral capillary loops, resulting in an early focal proliferative lesion (masson trichrome stain, ×200).
		Claude	The histologic changes are quite similar to Image 1, again depicting diffuse proliferative lupus nephritis. Endocapillary hypercellularity is extensive, seen in virtually all glomeruli, indicating an active class IV lesion. Wirelope capillary walls are readily identified, further supporting the diagnosis of lupus nephritis. No prominent tubular or interstitial changes are seen at this magnification. The histologic features corroborate diffuse proliferative lupus nephritis, class IV.
3		Standard pathological description	IgA nephropathy. There is diffuse mesangial hypercellularity with segmental endocapillary hypercellularity and occasional peripheral basement membrane duplication, evidence of deposits extending to peripheral loops (Jones silver stain, ×200).
		Claude	This medium power view again demonstrates florid diffuse proliferative glomerulonephritis. Marked expansion of the mesangium and endocapillary proliferation are resulting in lobular accentuation of the tufts. Abundant immune deposits are seen as large, chunky subendothelial and mesangial deposits, staining fuchsinophilic on this trichrome stain. No crescents or necrosis are definitely identified in this single section. Tubular, interstitial and vascular details are obscured at this magnification. The changes are diagnostic of diffuse proliferative lupus nephritis, ISN/RPS class IV-G.
4		Standard pathological description	IgA nephropathy. massive deposits are visualized as periodic acid－schiff-positive areas within the mesangium in this case of advanced iga nephropathy with mesangial hypercellularity and segmental sclerosis (top upper right) (Jones silver stain, ×400).
		Claude	The biopsy exhibits similar findings to the prior images - diffuse proliferative glomerulonephritis with marked hypercellularity obliterating the capillary lumina and causing lobular accentuation. Scattered fuchsinophilic immune deposits are highlighted on trichrome stain. A single small cellular crescent is noted in one glomerulus, but no frank necrosis is appreciated. Tubules are focally atrophic but interstitial fibrosis appears minimal in the sampled tissue. Again, these features are diagnostic of active diffuse lupus nephritis, ISN/RPS class IV.
5		Standard pathological description	IgA nephropathy. The underlying iga nephropathy giving rise to the crescentic injury is evident by moderate mesangial hypercellularity with segmental peripheral capillary loop alteration (right). in addition, characteristic iga positivity by immunofluorescent and electron microscopic demonstration of well-defined deposits were present (Jones silver stain, ×200).
		Claude	This final image demonstrates identical histology to that in the previous lupus nephritis biopsies. Marked diffuse proliferation effaces the glomerular architecture and forms fuchsinophilic wireloop lesions and scattered crescents. No normal-appearing glomeruli are present. The surrounding parenchyma shows mild patchy tubular atrophy without significant interstitial inflammation or fibrosis. The histologic features are classic for active diffuse proliferative lupus nephritis, class IV-G (A).
6	IgG4-related tubulointerstitial nephritis	Standard pathological description	IgG4-related tubulointerstitial nephritis. there is an expansile tubulointerstitial nephritis with lymphoplasmacytic infiltrate and tubular atrophy. glomeruli are well-preserved in this case (hematoxylin and eosin, ×100).
		Claude	This kidney section shows diffuse mesangial hypercellularity with increased mesangial matrix. The glomerular capillary loops appear patent. There is minimal interstitial fibrosis or tubular atrophy. The findings are consistent with mesangial proliferative glomerulonephritis, possibly IgA nephropathy or lupus nephritis class II. Further studies with immunofluorescence and electron microscopy would help clarify the specific diagnosis.
7		Standard pathological description	IgG4-related tubulointerstitial nephritis. there is advanced fibrosis with a whorling expansile fibrotic process and scattered plasma cells, with sparing of glomeruli (Jones silver stain, ×200).
		Claude	The kidney tissue demonstrates membranoproliferative changes in the glomeruli. There is global endocapillary hypercellularity with lobular accentuation of the glomerular tufts. Double contour formation of the capillary loops is evident. The interstitium shows mild fibrosis. Tubules are relatively intact. The features suggest membranoproliferative glomerulonephritis type 1. Correlation with immunofluorescence and clinical findings is recommended.
8		Standard pathological description	IgG4-related tubulointerstitial nephritis. Expansile interstitial nephritis with frequent plasma cells and whorling pattern of fibrosis are evident, with sparing of glomeruli (Jones silver stain, ×200).
		Claude	Examination of this renal biopsy reveals diffuse global membranous thickening of the glomerular capillary walls. Spikes are seen projecting from the glomerular basement membrane on silver stain. Podocytes appear prominent. Tubular atrophy and interstitial fibrosis are mild. No crescents or necrotizing lesions are identified. The morphology is diagnostic of membranous glomerulonephritis (MGN). Staging and subtyping of MGN requires electron microscopy. Excluding secondary causes clinically is advised.
9	proliferative glomerulonephritis with monoclonal deposits (PGNMID)	Standard pathological description	proliferative glomerulonephritis with monoclonal deposits (PGNMID). There is diffuse and global endocapillary hypercellularity in PGNMID (periodic acid－schiff, ×400).
		Claude	This renal biopsy shows diffuse thickening of the glomerular basement membrane with spike-like protrusions, giving a "spiky" appearance. The capillary lumens are patent. There is no significant hypercellularity or mesangial expansion. The tubules and interstitium appear unremarkable. These findings are consistent with membranous nephropathy (MN). Special stains like Jones silver would help confirm the basement membrane changes. The pathogenesis of MN often involves immune complex deposition, so immunofluorescence studies could provide additional insight.
10		Standard pathological description	proliferative glomerulonephritis with monoclonal deposits (PGNMID). There is endocapillary hypercellularity, and also small cellular crescent (periodic acid－schiff, ×400).
		Claude	The glomeruli in this biopsy demonstrate marked hypercellularity with an increase in mesangial matrix and endocapillary proliferation, resulting in the obliteration of capillary lumens in some areas. A few neutrophils are present within the glomerular tufts. The tubules show mild atrophy, and the interstitium has a patchy chronic inflammatory infiltrate. Overall, the features suggest a proliferative glomerulonephritis, possibly post-infectious or related to a systemic disease like lupus nephritis. Further evaluation with immunofluorescence and electron microscopy would help narrow the differential diagnosis.
11		Standard pathological description	proliferative glomerulonephritis with monoclonal deposits (PGNMID). Global endocapillary hypercellularity is evident, with frequent but segmental cellular interposition (periodic acid－schiff, ×400).
		Claude	This biopsy reveals glomeruli with markedly dilated capillary loops forming "lobules." The lobules are separated by an expanded mesangial matrix with a nodular appearance. Some nodules have a laminated, "onion skin" appearance. The tubules appear atrophic, and there is interstitial fibrosis. These changes are diagnostic of nodular diabetic glomerulosclerosis (Kimmelstiel-Wilson lesion), a manifestation of advanced diabetic nephropathy. The extent of glomerulosclerosis and tubulointerstitial scarring indicates significant chronic kidney injury. Correlation with clinical history and laboratory findings would provide a more comprehensive assessment.
12	Autosomal dominant polycystic kidney disease	Standard pathological description	Autosomal dominant polycystic kidney disease. Microscopic examination reveals the cysts to be lined by flattened epithelium and the interstitial tissue to consist predominately of fibrous tissue with small capillaries and atrophic tubules (hematoxylin and eosin, ×400).
		Claude	The histological section shows renal cortical tissue with numerous glomeruli distributed throughout. The glomeruli appear enlarged and hypercellular, indicating a proliferative glomerulonephritis. There is marked mesangial expansion and increased mesangial matrix deposition. The capillary lumens are narrowed due to endocapillary proliferation. Some glomeruli show segmental sclerosis and adhesions to the Bowman's capsule. The tubules are mostly intact, with focal areas of tubular atrophy and interstitial fibrosis. The blood vessels show mild arteriosclerosis. Overall, the findings are consistent with a proliferative glomerulonephritis, likely membranoproliferative glomerulonephritis (MPGN).
13		Standard pathological description	Autosomal dominant polycystic kidney disease. Only a minority of the nephrons develop cysts. normal tubular structures are scattered throughout the interstitial fibrous tissue (hematoxylin and eosin, ×200).
		Claude	This section also demonstrates renal cortical tissue, but with more advanced pathological changes compared to Image 1. The glomeruli are markedly enlarged and show diffuse mesangial expansion and hypercellularity. There is extensive endocapillary proliferation resulting in obliteration of capillary lumens. Many glomeruli exhibit crescent formation, indicating rapidly progressive glomerulonephritis. The tubules show widespread atrophy and loss, with extensive interstitial inflammation and fibrosis. Blood vessels have moderately thickened walls due to hypertensive changes. These histological features are suggestive of a crescentic glomerulonephritis, possibly due to ANCA-associated vasculitis or lupus nephritis.

14	henoch–schönlein purpura nephritis	Standard pathological description	henoch–schönlein purpura nephritis. The glomerulus demonstrates moderate diffuse mesangial proliferation with an early epithelial crescent (periodic acid–schiff, ×400).
		Claude	This slide shows kidney tissue with numerous variably-sized cystic structures lined by cuboidal epithelium. The cysts appear to be diffusely distributed throughout the cortex and medulla. The intervening parenchyma demonstrates some atrophy and fibrosis. There are no overtly dysplastic changes appreciated. The histologic features are consistent with adult polycystic kidney disease (APKD).
15		Standard pathological description	henoch–schönlein purpura nephritis. The glomerulus shows evidence of lobular accentuation, mesangial hypercellularity, and focal thickening of the peripheral capillary walls and segmental necrosis (hematoxylin and eosin, ×400).
		Claude	The kidney tissue in this image demonstrates similar findings to Image 1, with numerous cysts of varying size diffusely involving the parenchyma. The cysts are lined by a simple cuboidal epithelium. Some of the cysts contain proteinaceous material. The surrounding kidney tissue shows atrophic changes and interstitial fibrosis. Again, the morphologic findings are in keeping with APKD.
16		Standard pathological description	henoch–schönlein purpura nephritis. There is segmental necrosis with accumulation of fibrinoid material. The remainder of the glomerulus shows mesangial hypercellularity and an increase in matrix (trichrome, ×400).
		Claude	This higher magnification view provides a closer look at the cystic changes in APKD. Variably sized cysts are seen, some with eosinophilic proteinaceous material in the lumina. The cyst epithelial lining consists of a simple cuboidal epithelium without overt atypia. The intervening kidney parenchyma is atrophic with interstitial fibrosis.
17		Standard pathological description	henoch–schönlein purpura nephritis. There is an adhesion to Bowman's capsule, early crescent formation, and an increase in mesangial matrix.(Jones, ×400).
		Claude	As with the prior images, this slide demonstrates cystic change diffusely involving the kidney, consistent with APKD. Multiple simple cysts are appreciated, showing no overtly complex architecture or epithelial atypia. Some cysts contain eosinophilic proteinaceous material. The background kidney parenchyma is atrophic.
18		Standard pathological description	henoch–schönlein purpura nephritis. segmental sclerosis is prominent with loss of the normal architecture. The remainder of the glomerulus appears to have somewhat less hypercellularity (trichrome, ×400).
		Claude	This final image reveals histologic findings similar to the previous slides, all demonstrating pathologic features of APKD. There are numerous simple cysts lined by cuboidal epithelium diffusely distributed throughout the kidney tissue. Eosinophilic cyst contents are noted. The non-cystic kidney tissue is atrophic with interstitial fibrosis.
19	acute postinfectious glomerulonephritis	Standard pathological description	acute postinfectious glomerulonephritis. There is diffuse, global exudative proliferative appearance with prominent endocapillary hypercellularity and numerous neutrophils. There is also surrounding inflammation in the tubulointerstitium (periodic acid–schiff, ×400).
		Claude	This slide shows a section of kidney tissue with extensive tubular necrosis and interstitial inflammation. The tubules are dilated and lined by flattened epithelium, with some containing proteinaceous casts. There is marked interstitial edema and a diffuse infiltrate of lymphocytes and plasma cells. The glomeruli appear largely unaffected. These findings are consistent with acute tubular necrosis, likely secondary to ischemic or toxic injury.
20		Standard pathological description	acute postinfectious glomerulonephritis. The numerous polymorphonuclear leukocytes filling capillary lumens are clearly demonstrated, along with endocapillary hypercellularity and segmental cellular interposition (Jones silver stain, ×400).
		Claude	The kidney tissue in this slide demonstrates similar changes to Image 1, with prominent tubular injury, interstitial edema, and inflammation. However, the inflammatory infiltrate appears denser and composed predominantly of neutrophils, suggesting an infectious etiology such as acute pyelonephritis. The glomeruli remain relatively spared. Further clinical correlation and cultures would be helpful to confirm the underlying cause.
21		Standard pathological description	acute postinfectious glomerulonephritis. endocapillary hypercellularity and numerous polymorphonuclear leukocytes both within capillary loops and within the mesangial area are present. Deposits are not visualized here, although they may occasionally be seen by light microscopy (hematoxylin and eosin, ×1000).
		Claude	In this renal biopsy, the glomeruli show diffuse hypercellularity with an increased number of mesangial and endothelial cells. The capillary loops are thickened and demonstrate "wire-loop" lesions, characteristic of membranoproliferative glomerulonephritis. There is also prominent tubular atrophy and interstitial fibrosis, indicating chronicity. Immunofluorescence and electron microscopy studies would help further subclassify this glomerulonephritis.
22		Standard pathological description	acute postinfectious glomerulonephritis. in the latter stages following onset of the disease, there may be less prominent neutrophilic infiltrate with focal segmental mesangial and/or endocapillary hypercellularity. immunofluorescence and electron microscopy studies are then key in pointing to a postinfectious etiology (periodic acid–schiff, ×200).
		Claude	This high-power view of a glomerulus reveals marked hypercellularity with numerous intracapillary inflammatory cells, likely neutrophils, leading to a "crescent" formation. This crescentic glomerulonephritis is often associated with rapidly progressive glomerulonephritis (RPGN). The surrounding tubules show acute injury and the interstitium is edematous with mixed inflammation. Urgent clinical correlation and serologic workup for ANCA, anti-GBM antibodies, and complement levels are indicated.
23	Acute interstitial nephritis	Standard pathological description	Acute interstitial nephritis. Higher power than Fig. 5.20 demonstrates the presence of mononuclear cells throughout the interstitium; the tubule lumina are relatively free of leukocytic infiltrate (hematoxylin and eosin, ×200).
		Claude	This slide shows diffuse proliferation and infiltration of lymphoid cells amidst kidney tissue. The lymphoid cells have large, atypical nuclei and scant cytoplasm, consistent with a high-grade lymphoma involving the kidney. The normal kidney architecture is largely effaced. This degree of tumor infiltration would significantly impair renal function.
24		Standard pathological description	Acute interstitial nephritis. there is a diffuse interstitial infiltrate with evidence of interstitial edema. glomeruli are relatively well preserved (hematoxylin and eosin, ×100).
		Claude	The pathology is similar to Image 1, with sheets of malignant lymphoid cells infiltrating and obscuring the renal parenchyma. However, a few round structures that likely represent residual glomeruli can still be discerned, suggesting slightly less extensive involvement compared to Image 1. The lymphoma cells again have enlarged, pleomorphic nuclei and minimal cytoplasm.
25		Standard pathological description	Acute interstitial nephritis. the mononuclear infiltrate is accompanied by abundant eosinophils and may have a granulomatous appearance (hematoxylin and eosin, ×400).
		Claude	Diffuse infiltration by atypical lymphocytes is again seen, compatible with high-grade lymphoma. Compared to the previous images, the tumor cells appear slightly smaller and more monomorphic, but still show large nuclei relative to the scant cytoplasmic rims. Occasional foci with preserved tubular outlines are noted. The tumor burden and pattern appears comparable to Image 2.
26	Acute tubular injury	Standard pathological description	Acute tubular injury. in acute tubular injury, there is dilation of the tubules and interstitial edema. in addition, the proximal tubules show evidence of loss of the brush border with blebbing of the apical membrane into the tubular lumen (hematoxylin and eosin, ×100).
		Claude	This slide shows renal tissue with an abnormal appearance. There is significant distortion of the normal kidney architecture. The glomeruli, which are the small filtering units of the kidney, appear enlarged and have a lobular appearance. Many glomeruli show segmental sclerosis, meaning portions of the glomeruli are scarred and no longer functional. The surrounding tubules also appear dilated and atrophic. The interstitium shows fibrosis and a chronic inflammatory infiltrate. Overall, this pattern is consistent with focal segmental glomerulosclerosis (FSGS), a disease process that can lead to impaired kidney function over time.
27		Standard pathological description	Acute tubular injury. Focal calcification of the tubular epithelium is also seen with evidence of denudation of the basement membrane (hematoxylin and eosin, ×200).
		Claude	This renal tissue specimen also demonstrates abnormalities in the glomeruli and tubules. The glomeruli are hypercellular, meaning they contain increased numbers of cells, particularly mesangial cells. Some glomeruli show a crescent-shaped collection of cells in the Bowman's space. The tubules appear atrophic with flattened epithelium. A lymphocytic inflammatory infiltrate is present in the interstitium. These findings suggest a proliferative glomerulonephritis, possibly crescentic glomerulonephritis, which can occur in various autoimmune or inflammatory conditions affecting the kidneys.
28		Standard pathological description	Acute tubular injury. there are fragments of cells within the tubular lumina. Flattening of the tubular epithelium, loss of nuclei, and a marked interstitial infiltrate with occasional inflammatory cells are also present (hematoxylin and eosin, ×200).
		Claude	The glomeruli in this sample show marked hypercellularity with an increase in mesangial matrix and mesangial cells. Capillary walls appear thickened. Some glomeruli exhibit accentuated lobulation. Tubules are dilated and contain eosinophilic proteinaceous material, likely due to protein leakage from damaged glomeruli. There is moderate interstitial fibrosis and tubular atrophy. This pattern suggests an advanced stage of membranoproliferative glomerulonephritis (MPGN). MPGN has multiple potential causes including infections, autoimmune diseases, and paraproteinemias.
29		Standard pathological description	Acute tubular injury. Proteinaceous casts are present in the distal tubules made up of t amm–Horsfall protein (hematoxylin and eosin, ×400).
		Claude	This high magnification view of a glomerulus demonstrates diffuse thickening of the capillary walls and mesangial matrix expansion. The capillary lumens are narrowed. No significant hypercellularity is seen. Eosinophilic deposits are noted along the peripheral capillary walls, giving a "wire-loop" appearance. The tubules appear mildly dilated. Mild interstitial inflammation is present. These features are suggestive of membranous glomerulonephritis, a common cause of nephrotic syndrome in adults. The immune complex deposits along the subepithelial aspect of the basement membrane correspond to the "wire-loop" lesions seen by light microscopy.

30	Acute pyelonephritis	Standard pathological description	Acute pyelonephritis. Low-power image of a renal biopsy showing diffuse interstitial infiltrate that is predominately polymorphonuclear and leukocytic. there is interstitial edema; some tubular atrophy and leukocytes can be seen in the lumen of the tubules (hematoxylin and eosin, ×100).
		Claude	This slide shows kidney tissue with numerous glomeruli present. The glomeruli appear enlarged and hypercellular, with increased mesangial matrix and endocapillary proliferation resulting in a lobular appearance. The capillary lumens are narrowed. The surrounding tubules show evidence of atrophy and protein reabsorption droplets. The interstitium has a patchy lymphocytic infiltrate. These findings are consistent with a proliferative glomerulonephritis, possibly membranoproliferative glomerulonephritis (MPGN).
31		Standard pathological description	Acute pyelonephritis with abscess formation. the abscesses are frequently glomerulocentric and are destructive of the renal parenchyma (hematoxylin and eosin, ×200).
		Claude	This high magnification view demonstrates markedly enlarged, lobular glomeruli with obliterated capillary lumens, a finding known as a "tram-track" appearance. There is prominent endocapillary hypercellularity with numerous inflammatory cells present. The mesangial areas are expanded by increased matrix and cells. Crescents and fibrinoid necrosis are not clearly evident in this view. These features support a diagnosis of an aggressive proliferative glomerulonephritis, again consistent with MPGN or a related entity.
32		Standard pathological description	Acute pyelonephritis. there is a diffuse interstitial infiltrate with polymorphonuclear leukocytes (hematoxylin and eosin, ×200).
		Claude	The slide shows similar findings to Image 2, with enlarged glomeruli demonstrating global endocapillary hypercellularity and lobular accentuation of the glomerular tufts. Obliterated capillary loops and increased mesangial matrix are seen. A cellular crescent is present in one glomerulus at the 7 o'clock position. Mixed interstitial inflammation is noted. The overall picture indicates a proliferative and crescentic glomerulonephritis, possibly representing a more advanced stage of MPGN or an overlapping disease process.
33		Standard pathological description	Acute pyelonephritis. Not only is infiltration of polymorphonuclear leukocytes present within the interstitium and in the tubular lumens, but evidence of tubulitis also is seen (hematoxylin and eosin, ×400).
		Claude	This high power view highlights a glomerulus with marked endocapillary proliferation, a cellular crescent filling Bowman's space, and segmental fibrinoid necrosis disrupting the glomerular basement membranes. The capillary lumens are difficult to discern. A mixed interstitial infiltrate is present. These are features of a severe crescentic glomerulonephritis with a proliferative component. Rapidly progressive glomerulonephritis, vasculitis, or lupus nephritis would be in the differential diagnosis.
34	Focal segmental glomerulosclerosis	Standard pathological description	Focal segmental glomerulosclerosis (FSGS). The typical segmental sclerotic lesion in FsGs is characterized by increased matrix and obliteration of capillary lumina, frequently with hyalinosis and adhesions, as illustrated here. There is surrounding tubulointerstitial fibrosis. The uninvolved segment of the glomerulus appears unremarkable (Jones silver stain, ×200).
		Claude	This slide shows a glomerulus, the filtration unit of the kidney, at high magnification. The glomerulus appears enlarged and hypercellular, with an increased number of cells in the mesangial matrix and capillary loops. This suggests a proliferative glomerulonephritis, possibly membranoproliferative glomerulonephritis (MPGN) given the lobular accentuation of the glomerular tuft. Further special stains and clinical correlation would be needed for a definitive diagnosis.
35		Standard pathological description	Focal segmental glomerulosclerosis (FSGS). Near end-stage FsGs is present, with global or near global sclerosis of all glomeruli and extensive tubulointerstitial fibrosis and vascular thickening (Jones silver stain, ×200).
		Claude	This glomerulus also demonstrates hypercellularity and increased mesangial matrix, consistent with a proliferative glomerulonephritis. The capillary walls appear thickened, which could indicate an membranous component as well. The surrounding tubules look unremarkable. Again, MPGN would be a key differential diagnosis to consider.
36		Standard pathological description	Focal segmental glomerulosclerosis (FSGS). an advanced segmental sclerotic lesion of FSGS is shown, with only minimal hyaline droplets. There is increased mesangial matrix and obliteration of capillary lumina involving the majority of the glomerulus. The uninvolved portion of the glomerulus has mild increase in mesangial matrix. The adjacent tubule shows atrophy and a proteinaceous cast (periodic acid–schiff, ×400).
		Claude	Here we see a glomerulus with an enlarged, hypercellular tuft showing accentuated lobulation. The mesangial matrix is expanded. Capillary loops are thickened and appear obliterated in areas. Surrounding tubules are largely unremarkable. The findings are consistent with a membranoproliferative pattern of injury. Correlation with immunofluorescence and electron microscopy would help further subclassify the MPGN.
37		Standard pathological description	Focal segmental glomerulosclerosis (FSGS). in this case of FsGs, there was extensive hyalinosis in the sclerotic areas, which are characterized by increased mesangial matrix and obliteration of capillary lumina. There are also adhesions of the sclerotic segments to Bowman' s capsule, with thickened and disrupted Bowman' s capsule. The hyalinosis represents an insudation of plasma proteins, reflecting endothelial injury (Jones silver stain, ×400).
		Claude	This glomerulus shows marked enlargement and hypercellularity with an increase in mesangial matrix and cellularity. Capillary walls are thickened, with some appearing eosinophilic and rigid, suspicious for wire-loop lesions. A membranoproliferative pattern is evident. In the context of suspected wire-loops, lupus nephritis, specifically class IV (diffuse proliferative) would be high on the differential. Further workup with special stains, immunofluorescence and serology is warranted.
38	anti-glomerular basement membrane antibody–mediated glomerulonephritis	Standard pathological description	anti-glomerular basement membrane (GBM) antibody–mediated glomerulonephritis. There is evident segmental necrosis in both glomeruli, with uninvolved segments of the glomeruli showing no proliferation or evidence of immune complexes. There is early cellular crescent formation (Jones silver stain, ×100).
		Claude	This slide shows kidney tissue with extensive glomerular changes. The glomeruli appear enlarged and hypercellular, with thickening of the capillary walls and mesangial matrix expansion. There is also increased lobulation of the glomerular tufts. These findings are consistent with a membranoproliferative pattern of glomerulonephritis. The surrounding tubules and interstitium appear unremarkable in this field.
39		Standard pathological description	anti-glomerular basement membrane (GBM) antibody–mediated glomerulonephritis. There is fibrinoid necrosis with karyorrhexis and ruptured fragments of GBM, with a small remaining intact portion of the glomerulus at the top. There is surrounding cellular crescent formation, and periglomerular inflammatory infiltrate (Jones silver stain, ×400).
		Claude	Similar to the previous image, this slide demonstrates diffuse glomerular involvement with a membranoproliferative pattern. The glomeruli are enlarged with hypercellularity, capillary wall thickening, and mesangial expansion. Some of the glomeruli also show accentuated lobulation. No significant tubular or interstitial pathology is evident in the field shown.
40		Standard pathological description	anti-glomerular basement membrane (GBM) antibody–mediated glomerulonephritis. early segmental fibrinoid necrosis is present, with GBM rupture. There is not yet a cellular crescentic reaction. The remaining portion of the glomerulus shows no proliferation or evidence of immune complexes (Jones silver stain, ×400).
		Claude	This high power view highlights the glomerular changes in more detail. The glomerular tuft is enlarged and hypercellular with numerous endocapillary cells, likely including both native glomerular cells and infiltrating inflammatory cells. The capillary walls are thickened with splitting of the basement membranes, giving a "tram-track" appearance in some loops, consistent with a membranoproliferative pattern of injury.
41		Standard pathological description	anti-glomerular basement membrane (GBM) antibody–mediated glomerulonephritis. in this more advanced lesion, there is corrugation and rupture of GBM with early fibrocellular organization of the crescent and rupture of Bowman' s capsule with corresponding fibrinoid necrosis and surrounding periglomerular inflammation (Jones silver stain, ×400).
		Claude	In this final image, the membranoproliferative pattern affecting the glomeruli is again appreciated. The marked glomerular enlargement and hypercellularity with thickened, split capillary walls is evident. Some crescentic changes may be developing in one of the glomeruli, with apparent early cellular crescent formation, suggesting a rapidly progressive component of the disease process.
42	Chronic interstitial fibrosis and tubular atrophy	Standard pathological description	Chronic interstitial fibrosis and tubular atrophy IF 2TA2. There are definite areas of interstitial scarring with prominent tubular atrophy. The scarring can occupy 25–50% of the cortex (hematoxylin and eosin, ×100).
		Claude	This kidney pathology slide shows glomeruli with mesangial and endocapillary hypercellularity, indicating active proliferative changes. The capillary walls appear thickened with split basement membranes, suggesting membranoproliferative glomerulonephritis (MPGN). There is also evidence of focal segmental glomerulosclerosis (FSGS) with segmental consolidation and adhesions to Bowman's capsule. The tubules show mild atrophy and the interstitium has patchy mononuclear inflammatory infiltrates. Overall, the findings are consistent with an active proliferative glomerulonephritis, likely MPGN, with secondary FSGS changes.
43		Standard pathological description	Chronic interstitial fibrosis and tubular atrophy IF1 TA1. There is focal interstitial scarring associated with minimal tubular atrophy occupying less than 25% of the cortex (hematoxylin and eosin, ×100).
		Claude	Similar to Image 1, this kidney biopsy demonstrates glomeruli with marked mesangial and endocapillary hypercellularity. The capillary walls are thickened with double contours, indicating MPGN. Focal segments of glomeruli show consolidation and adhesions to Bowman's capsule, in keeping with secondary FSGS. The tubulointerstitium has mild tubular atrophy and patchy inflammation. These changes support a diagnosis of MPGN with superimposed FSGS.
		Standard pathological description	chronic pyelonephritis shows a nonspecific interstitial infiltrate, predominately with lymphocytes. the parenchyma is diffusely infiltrated (hematoxylin and eosin, ×200).

44	chronic pyelonephritis	Claude	This kidney biopsy demonstrates diffuse proliferative glomerulonephritis. The glomeruli show marked global endocapillary hypercellularity with obliteration of capillary lumens. There is also prominent mesangial hypercellularity and matrix expansion. Several glomeruli have a lobular appearance. The tubules appear mildly atrophic with focal infiltration by mononuclear inflammatory cells. These findings are consistent with an acute proliferative glomerulonephritis, possibly post-infectious or related to lupus nephritis (ISN/RPS class IV). Further workup with immunofluorescence and electron microscopy would help differentiate the etiology.
45		Standard pathological description	chronic pyelonephritis manifests a nonspecific interstitial infiltrate, predominately with lymphocytes. there is evidence of destruction of the renal parenchyma with atrophy of tubules (hematoxylin and eosin, ×200).
		Claude	The glomeruli in this biopsy show similar changes to Image 1, with diffuse endocapillary and mesangial hypercellularity resulting in a lobular configuration. The capillary walls appear thickened, and occasional double contours (tram-tracking) are noted, suggestive of membranoproliferative glomerulonephritis (MPGN). The tubulointerstitium shows mild tubular atrophy and focal interstitial inflammation. The findings support a diagnosis of an MPGN pattern of injury, which could be primary (idiopathic) or secondary to systemic diseases like lupus, chronic infections, or paraproteinemias. Additional studies are needed to determine the specific subtype and guide treatment.
46	Diffuse mesangial sclerosis	Standard pathological description	Diffuse mesangial sclerosis with typical appearance of dense expansion of collagenous material within mesangium with minimal increase in mesangial cellularity. There is associated tubular atrophy and interstitial fibrosis (masson trichrome stain, ×400).
		Claude	This slide shows a section of kidney tissue with numerous round, purple-stained structures which appear to be glomeruli. The glomeruli look enlarged and have an increased cellularity, suggesting a proliferative glomerulonephritis. The surrounding tubules and interstitium also show some areas of purple staining, possibly indicating inflammation or fibrosis. Overall, the findings are consistent with a diffuse proliferative glomerulonephritis pattern of injury.
47		Standard pathological description	Diffuse mesangial sclerosis. Glomeruli show extensive sclerosis, with small, shrunken appearance and dense expansion of mesangial matrix, blue on trichrome stain, with little increase in mesangial cellularity. There is associated proportional tubular atrophy and interstitial fibrosis. occasional dilated tubules may be present (masson trichrome stain, ×100).
		Claude	Similar to the first image, this slide demonstrates diffusely affected glomeruli with hypercellularity and an increased amount of mesangial matrix, staining purple. The glomeruli appear lobulated. There are also scattered foci of tubular atrophy and interstitial inflammation. The blood vessels look unremarkable. The pattern of injury is consistent with a membranoproliferative glomerulonephritis.
48		Standard pathological description	Diffuse mesangial sclerosis with sclerosis of glomeruli, with small, shrunken appearance, with dense sclerosis obliterating the tuft and associated tubular atrophy and interstitial fibrosis. occasional tubules show mild dilation (Jones silver stain, ×200).
		Claude	This high magnification view focuses on a single glomerulus with prominent lobulation and hypercellularity, again demonstrating a membranoproliferative pattern of injury as in Image 2. The capillary loops are thickened and have a "split" appearance, suggesting possible membranoproliferative glomerulonephritis Type I. Some red blood cell casts are seen in the tubular lumina, indicating active glomerular inflammation.
49	membranoproliferative glomerulonephritis	Standard pathological description	membranoproliferative glomerulonephritis (MPGN). mpGN is characterized by diffuse endocapillary hypercellularity, which results in a lobular, uniform appearance of glomeruli (periodic acid–schiff, ×100).
		Claude	This slide shows a section of renal tissue with numerous glomeruli (the small round structures). The glomeruli appear enlarged and have thick, hypercellular walls, which could indicate membranoproliferative glomerulonephritis (MPGN). There is also increased mesangial matrix and cellularity. The surrounding tubules look fairly unremarkable. Overall, the pattern is consistent with a proliferative glomerular disease process.
50		Standard pathological description	membranoproliferative glomerulonephritis (MPGN). There is less marked endocapillary hypercellularity, with widespread double contours of the glomerular basement membrane, so-called tram-tracking (Jones silver stain, ×400).
		Claude	Similar to the first image, this slide demonstrates glomeruli with markedly thickened, hypercellular walls. The glomerular tufts nearly fill the Bowman's space. Some of the tubules appear dilated. The prominent lobular pattern of the glomeruli, along with the hypercellularity, supports a diagnosis of MPGN, likely type 1. Further special stains could help confirm the specific type.
51		Standard pathological description	membranoproliferative glomerulonephritis (MPGN). in some cases of mpGN, there may be nodular glomerulosclerosis and massive deposits. occasional polymorphonuclear leukocytes are also present, in addition to the mesangial and endocapillary proliferation. a small incipient cellular crescent is present. These morphologic features suggest the possibility of a secondary etiology of the mpGN lesion (Jones silver stain, ×400).
		Claude	Again, this renal tissue has numerous glomeruli with the same hypercellular, thickened appearance as in the previous images. The membranoproliferative pattern is evident. Some tubules are dilated and contain proteinaceous material, suggesting proteinuria as a result of the glomerular damage. There are no obvious crescents or necrosis. The interstitium looks mildly expanded, possibly due to edema or early fibrosis.
52	arterionephrosclerosis	Standard pathological description	arterionephrosclerosis. There is severe hyalinosis of this interlobular artery, with swollen endothelial cells and a few red blood cell fragments, indicative of early, accelerated endothelial injury (Jones silver stain, ×200).
		Claude	This slide shows a kidney glomerulus with significant mesangial hypercellularity and matrix expansion, consistent with mesangial proliferative glomerulonephritis. The capillary lumens appear narrowed. There is no evidence of crescent formation or necrosis.
53		Standard pathological description	arterionephrosclerosis. Moderate to severe hyalinosis is present in this arteriole. it is cut multiple times in this section, because of its tortuosity. The hyalinosis is eccentric (hematoxylin and eosin, ×200).
		Claude	This glomerulus demonstrates similar changes to Image 1, with marked mesangial hypercellularity and increased matrix material expanding the mesangial regions and compressing the capillary lumens. A few capillary loops appear completely collapsed. The surrounding tubules look unremarkable. The findings suggest mesangioproliferative glomerulonephritis.
54		Standard pathological description	arterionephrosclerosis. There is intimal fibrosis, thickening of the media, and increased thickness of the adventitia (periodic acid–Schiff, ×100).
		Claude	Significant interstitial fibrosis and tubular atrophy can be seen in this image, with thick bands of fibrous tissue separating atrophic tubules. A sclerotic glomerulus is present as well. These chronic changes indicate advanced kidney disease, possibly end-stage renal disease or the late stages of severe glomerulonephritis leading to scarring.
55		Standard pathological description	arterionephrosclerosis. There is duplication of the intima, with intimal fibroplasia, resulting in a thicker, less compliant wall of this interlobular artery (periodic acid–Schiff, ×200).
		Claude	This high magnification view of a glomerulus shows basement membrane thickening and duplication along the capillary walls, forming wire loop lesions. Mesangial hypercellularity is noted. Occasional intracapillary hyaline thrombi are seen. This pattern is characteristic of membranoproliferative glomerulonephritis (MPGN) with cryoglobulinemia, which can be seen in various conditions like viral hepatitis C, autoimmune disorders, and lymphoproliferative diseases.
56	Medullary cystic disease	Standard pathological description	Medullary cystic disease. The cyst is lined by flattened epithelium and surrounded by some relatively normal–appearing tubular structures (hematoxylin and eosin, ×200).
		Claude	The slide shows a section of renal cortex with numerous glomeruli. The glomeruli appear largely normal in size and cellularity. However, there is marked thickening and "spike" formation of the glomerular basement membrane, characteristic of membranous glomerulonephritis. Some tubules contain proteinaceous casts. The interstitium shows only mild focal inflammation. Overall, the findings are consistent with membranous nephropathy.
57		Standard pathological description	end-stage medullary cystic disease. The cortex proximal to the cysts shows marked atrophy with interstitial scarring and glomerulosclerosis. There is also marked hyalin and arteriolosclerosis (hematoxylin and eosin, ×200).
		Claude	This slide also demonstrates a section of renal cortex. The glomeruli show significant mesangial expansion and increased mesangial matrix and cellularity. Many glomeruli have a lobular appearance. Capillary walls are thickened. Some tubules are atrophic with protein reabsorption droplets in the epithelial cells. A moderate interstitial inflammatory infiltrate is present. The changes are suggestive of diabetic glomerulosclerosis with a secondary focal interstitial nephritis.
58		Standard pathological description	Medullary cystic disease. The adjacent tubules show evidence of atrophy, and interstitial fibrosis is present. There is no significant interstitial infiltrate (hematoxylin and eosin, ×100).
		Claude	The biopsy here reveals diffuse glomerular abnormalities, with extensive mesangial expansion and nodule formation, giving the glomeruli a "lumpy-bumpy" appearance. Capillary lumens are narrowed. Tubular atrophy and interstitial fibrosis are present. Hyaline arteriolosclerosis is noted. This pattern is characteristic of advanced diabetic nephropathy, likely class III or IV. Clinical correlation with proteinuria, decreased GFR and other findings of long-standing diabetes would be expected.
59		Standard pathological description	Collapsing glomerulopathy. extensive collapse with marked visceral epithelial cell hyperplasia in collapsing glomerulopathy (Jones silver stain, ×400).
		Claude	This slide shows numerous renal glomeruli with mild to moderate mesangial hypercellularity and matrix expansion. The capillary walls appear mildly thickened, suggestive of membranous changes. No crescents or necrosis are seen. The tubules and interstitium appear unremarkable. These findings are consistent with an early membranoproliferative glomerulonephritis pattern of injury.

60	Collapsing glomerulopathy	Standard pathological description	Collapsing glomerulopathy. Collapsing glomerulopathy is characterized by collapse of the glomerular tuft with marked proliferation of overlying visceral epithelial cells, often with prominent protein droplets. The collapse may be global, or more segmental (Jones silver stain, ×400).
		Claude	The glomeruli in this section demonstrate similar changes to Image 1, with mesangial hypercellularity and matrix expansion, as well as mildly thickened capillary loops. However, the degree of involvement appears slightly more pronounced compared to the previous slide. The tubules and interstitium remain unremarkable. The overall pattern suggests a membranoproliferative glomerulonephritis, likely type 1.
61		Standard pathological description	Collapsing glomerulopathy. There is collapse of the glomerular tuft and overlying hyperplasia of the visceral epithelial cells, with prominent protein reabsorption droplets (Jones silver stain, ×400).
		Claude	This high-power view highlights the glomerular changes, with more evident mesangial hypercellularity and matrix accumulation. The capillary walls are thickened and appear rigid, with segmental double contours visible, indicative of membranous changes. No crescents, fibrinoid necrosis, or endocapillary hypercellularity are appreciated. The findings support a membranoproliferative glomerulonephritis with a membranous component.
62	Diabetic nephropathy	Standard pathological description	Diabetic nephropathy. The arteriolar hyalinosis in diabetes typically involves both afferent and efferent arterioles. The glomerulus shows diffuse mesangial matrix increase without formation of Kimmelstiel–Wilson nodules in this case. There is thickening of bowman' s capsule and a small "capsular drop," that is, hyalin within bowman' s capsule at the 12 o' clock position (periodic acid–Schiff, ×200).
		Claude	This slide shows kidney tissue with enlarged, irregularly shaped glomeruli displaying mesangial hypercellularity. There is also increased mesangial matrix deposition. The capillary loops appear thickened and narrowed. These findings are consistent with a mesangioproliferative glomerulonephritis pattern of injury.
63		Standard pathological description	Diabetic nephropathy. The lesions in diabetic nephropathy are characterized by arteriolar hyalinosis, mesangial matrix expansion, and glomerular basement membrane thickening. There is also associated tubular interstitial fibrosis (periodic acid–Schiff, ×100).
		Claude	The glomeruli in this kidney tissue sample demonstrate marked mesangial and endocapillary hypercellularity, causing obliteration of the capillary lumina in many areas. Mesangial matrix expansion is also prominent. Wire-loop lesions can be seen, which are thickened capillary walls with a rigid appearance. The features are suggestive of diffuse proliferative lupus nephritis (WHO class IV).
64		Standard pathological description	Diabetic nephropathy. Diabetic nephropathy may manifest either as diffuse mesangial increase , or with nodular glomerulosclerosis as in this case. There are multiple nodules of mesangial matrix, surrounded by a small rim of intact capillaries. The glomerular basement membrane is prominent. The expanded nodules show a lamellated appearance, thought to be due to repeated injury with mesangiolysis and exuberant repair responses laying down increased matrix, and scattered vascular lumina (periodic acid–Schiff, ×200).
		Claude	Kidney biopsy showing proliferative glomerulonephritis with crescent formation. Cellular crescents can be seen in multiple glomeruli, formed by proliferation of parietal epithelial cells in Bowman's space. The crescents appear to be compressing the glomerular tufts. Fibrinoid necrosis is also present. Findings raise consideration of pauci-immune crescentic glomerulonephritis.
65		Standard pathological description	Diabetic nephropathy. Diffuse mesangial matrix increase and basement membrane thickening are evident in this case of early diabetic nephropathy. a "capsular drop" is seen at the bottom of bowman' s capsule. There is moderate increase in mesangial matrix and cellularity, with surrounding tubulointerstitial fibrosis. There are no deposits, splitting, or irregularities of the glomerular basement membrane (Jones silver stain, ×200).
		Claude	Glomeruli with irregular thickening and splitting of glomerular basement membranes, resulting in double contour appearance of the capillary walls. Mesangial hypercellularity and matrix expansion are noted as well. Subendothelial deposits may be present. The morphologic features are consistent with membranoproliferative glomerulonephritis (MPGN) pattern.
66		Standard pathological description	Diabetic nephropathy. The large Kimmelstiel–Wilson nodules contain small red blood cell fragments and frayed, irregular mesangial matrix, evidence of early mesangiolysis (Jones silver stain, ×200).
		Claude	Biopsy demonstrating glomeruli with hyaline thrombi within dilated capillary loops, imparting a lobular appearance to the tufts. Mesangiolysis, or dissolution of the mesangial matrix, is seen in association with the microaneurysmal capillary dilation. Fibrinoid necrosis and cellular interposition are also present. These are characteristic findings of thrombotic microangiopathy, suggestive of hemolytic uremic syndrome (HUS) or thrombotic thrombocytopenic purpura (TTP).
67		Standard pathological description	Diabetic nephropathy. The lamellated appearance of the Kimmelstiel–Wilson nodule characteristic of the nodular sclerosis form of diabetic nephropathy is shown, along with arteriolar hyalinosis and surrounding tubulointerstitial fibrosis (Jones silver stain, ×200).
		Claude	This slide shows a section of renal tissue with diffuse mesangial hypercellularity and matrix expansion, leading to the obliteration of capillary lumens. The glomeruli appear lobulated with accentuated lobular architecture. There is also evidence of basement membrane thickening. These findings are consistent with membranoproliferative glomerulonephritis (MPGN). Further immunofluorescence and electron microscopy studies would help subclassify the type of MPGN.
68		Standard pathological description	Diabetic nephropathy. There is an area of mesangiolysis with foam cells with loss of matrix and breaks of attachments of the capillary loop. This loss of tethering of the mesangium is thought to give rise to microaneurysms. The glomerular basement membrane is also thickened, without evident deposits (Jones silver stain, ×400).
		Claude	The glomeruli in this biopsy demonstrate marked mesangial and endocapillary hypercellularity, with numerous infiltrating neutrophils and occasional karyorrhexis (fragmented neutrophil nuclei). Capillary walls appear thickened, and some capillary lumens are occluded. The overall appearance suggests acute post-infectious glomerulonephritis or an infection-related glomerulonephritis. Clinical correlation and immunofluorescence studies would be helpful for a more definitive diagnosis.
69		Standard pathological description	Diabetic nephropathy. a large area of mesangial injury with red blood cell fragments and frayed, lucentappearing mesangial matrix, indicative of mesangiolysis, are shown (Jones silver stain, ×400).
		Claude	This high-power view reveals a glomerulus with global endocapillary proliferation, characterized by the accumulation of infiltrating monocytes, neutrophils, and endothelial cells within the glomerular capillaries. The mesangium also appears expanded by matrix and hypercellularity. These changes are suggestive of a proliferative glomerulonephritis, possibly post-infectious or lupus nephritis. Additional clinical information, serological tests, and immunofluorescence studies would aid in further classification.
70		Standard pathological description	Diabetic nephropathy. There is an area of mesangiolysis with fraying of the mesangium and loss of attachment of the mesangial area to the peripheral capillary loop. red blood cell fragments are also present (Jones silver stain, ×1000).
		Claude	The glomerulus in this image exhibits advanced disease with global sclerosis and obliteration of the capillary tuft. There is marked mesangial matrix expansion and hypercellularity, with the formation of a segmental sclerotic lesion. These features are indicative of focal segmental glomerulosclerosis (FSGS) with significant chronic damage. The presence of extensive sclerosis suggests a poor prognosis. Correlation with the patient's clinical history, serum creatinine levels, and proteinuria would provide additional context.
71		Standard pathological description	preeclampsia. Glomerulus demonstrates lobular accentuation with mesangial proliferation. The glomerular capillaries are collapsed. occasional capillaries show microthrombi. The endothelial cells appear swollen (hematoxylin and eosin, ×400).
		Claude	This slide shows a section of kidney tissue with normal glomeruli surrounded by significant tubular necrosis and interstitial inflammation. The extensive presence of red blood cells in the tubular lumens is indicative of acute tubular injury or necrosis, likely due to ischemic or nephrotoxic insults. The interstitium shows edema and infiltration by inflammatory cells, predominantly lymphocytes. Overall, the findings are consistent with acute tubular necrosis accompanied by interstitial nephritis.
72		Standard pathological description	preeclampsia. Glomerulus demonstrates dilated glomerular capillaries, and the endothelial cells appear swollen to fill the capillary lumens (endotheliosis) (silver methenamine, ×400).
		Claude	The glomeruli in this kidney section display diffuse mesangial expansion and hypercellularity, with numerous open capillary loops. There is mild interstitial fibrosis and tubular atrophy noted. The mesangial changes are suggestive of early diabetic nephropathy or another glomerular disease process involving mesangial proliferation. Further clinical correlation and special stains could help differentiate the specific etiology.
73		Standard pathological description	preeclampsia. The glomerulus demonstrates a bloodless appearance. The capillary loops are collapsed and appear to be thickened largely as a result of endothelial cell swelling (hematoxylin and eosin, ×400).
		Claude	Prominent globally sclerotic glomeruli are seen throughout this kidney biopsy, indicating advanced chronic kidney disease. The remaining glomeruli show segmental sclerosis and hyalinosis. Extensive interstitial fibrosis and tubular atrophy are present, along with scattered lymphocytic inflammation. These chronic changes are consistent with late-stage kidney disease, possibly due to longstanding hypertension, diabetes, or other chronic insults.
74		Standard pathological description	preeclampsia. Glomerulus demonstrates marked endotheliosis. The capillary lumens are narrowed by swollen endothelial cells (hematoxylin and eosin, ×400).
	preeclampsia	Claude	This high-power view demonstrates a glomerulus with marked mesangial and endocapillary hypercellularity, causing obliteration of the capillary lumens. The expanded mesangial matrix and numerous inflammatory cells within the glomerular tuft are consistent with a proliferative glomerulonephritis. Differential diagnoses include post-infectious glomerulonephritis, IgA nephropathy, or lupus nephritis, among others. Immunofluorescence and electron microscopy studies would be helpful for a definitive diagnosis.

75		Standard pathological description	preeclampsia. Some glomerular capillaries are markedly dilated and there is evidence of mesangiolysis and focal necrosis (trichrome, ×400).
		Claude	The glomeruli in this section display marked lobular accentuation, with thickened capillary walls and segmental areas of endocapillary proliferation. Focal double contours of the glomerular basement membranes are noted, suggesting membranoproliferative changes. The tubulointerstitium shows moderate fibrosis and tubular atrophy. These findings raise the possibility of a membranoproliferative glomerulonephritis (MPGN) pattern, which could be seen in conditions like chronic hepatitis C, autoimmune diseases, or monoclonal gammopathies.
76		Standard pathological description	preeclampsia. The glomerulus demonstrates the same findings as in Fig. 4.57 with evidence of capillary thrombosis, thickening of the capillary walls, and endothelial cell swelling (trichrome, ×400).
		Claude	This renal biopsy section shows diffuse proliferative glomerulonephritis. There is global hypercellularity of the glomerular tufts with endocapillary proliferation and infiltration by inflammatory cells, likely representing an acute immune-complex mediated process. The surrounding tubules appear atrophic with interstitial fibrosis, suggesting some chronicity as well. Overall, the findings are consistent with an active lupus nephritis or post-infectious glomerulonephritis.
77		Standard pathological description	preeclampsia. in glomeruli not as severely involved, there is an increase in mesangial matrix and thickening of the capillary walls as a result of endothelial cell swelling (hematoxylin and eosin, ×200).
		Claude	The glomeruli in this section also demonstrate a diffuse proliferative pattern, with prominent endocapillary hypercellularity occluding the capillary lumens. Additionally, several glomeruli show cellular crescents, indicating severe glomerular injury. The tubules are atrophic with interstitial inflammation and fibrosis. Wire-loop lesions are noted, highly suggestive of active Class IV lupus nephritis. The crescent formation portends a worse prognosis.
78		Standard pathological description	preeclampsia. in glomeruli not as severely involved, there is an increase in mesangial matrix and thickening of the capillary walls as a result of endothelial cell swelling (trichrome, ×400).
		Claude	This biopsy reveals membranoproliferative glomerulonephritis (MPGN). The glomerular tufts show mesangial and endocapillary proliferation with thickening of the capillary walls, resulting in a lobular appearance. Immune deposits are likely present within the mesangium and along the basement membrane. A trichrome stain would help highlight if there is double-contour formation. The interstitium shows inflammation and fibrosis. MPGN has multiple etiologies including infections, autoimmune diseases, and paraproteinemias which would need to be investigated.
79	Fibrillary glomerulonephritis	Standard pathological description	Fibrillary glomerulonephritis. in our series, approximately one-third of cases showed crescents. in this case, there is associated moderate mesangial hypercellularity, an important feature indicating by light microscopy that this crescentic lesion is likely not pauci-immune crescentic glomerulonephritis or anti-glomerular basement membrane (GBm) antibody–mediated glomerulonephritis. periglomerular fibrosis and early adhesions are also evident (Jones silver stain, ×400).
		Claude	This slide shows a section of kidney tissue with pronounced histopathological changes. The glomeruli, the filtration units of the kidney, exhibit a nodular sclerotic pattern. The mesangial matrix is expanded, forming distinct nodules that compress the capillary loops. This morphology is consistent with nodular glomerulosclerosis, also known as Kimmelstiel-Wilson nodules, which are characteristic of advanced diabetic nephropathy. The surrounding tubules appear atrophic with thickened basement membranes. There is also evidence of interstitial fibrosis and tubular atrophy (IFTA). Overall, these findings suggest a diagnosis of diabetic nephropathy with significant chronic changes.
80		Standard pathological description	Fibrillary glomerulonephritis. a membranoproliferative pattern is evident with mesangial and endocapillary hypercellularity and basement membrane duplication and cellular interposition (Jones silver stain, ×400).
		Claude	This kidney biopsy demonstrates different pathological features compared to the first slide. The glomeruli show marked enlargement and hypercellularity. The capillary loops are distended and occluded by endocapillary proliferation, likely due to an inflammatory infiltrate. Some glomeruli also exhibit crescent formation, characterized by the accumulation of cells within Bowman's space. The crescents appear cellular, indicating an active disease process. The tubules show signs of injury, including epithelial flattening and luminal dilation. The interstitium is edematous with a moderate inflammatory infiltrate. These histological findings are consistent with a diagnosis of rapidly progressive glomerulonephritis (RPGN), possibly secondary to an immune-mediated etiology such as anti-GBM disease, ANCA-associated vasculitis, or lupus nephritis. Further clinical correlation and additional studies (e.g., immunofluorescence, electron microscopy) would be helpful to determine the specific underlying cause.
81	microscopic polyangiitis	Standard pathological description	microscopic polyangiitis. Glomerulus with segmental area of necrosis with leukocytoclasia (karyorrhexis). a periglomerular infiltrate of mononuclear cells is also present (hematoxylin and eosin, ×400).
		Claude	This slide shows a section of kidney tissue with extensive global glomerulosclerosis. The glomeruli appear largely sclerotic and fibrotic, with loss of normal architecture. There is significant hypercellularity and matrix expansion, indicating chronic injury and scarring. The tubules are also affected, showing atrophy and dilation. This pattern is consistent with advanced kidney disease, likely from a chronic progressive condition such as diabetes, hypertension, or glomerulonephritis.
82		Standard pathological description	microscopic polyangiitis. Glomerulus with global increase in mesangial cellularity and focal area of fibrinoid necrosis associated with a cellular crescent. Karyorrhexis and leukocytic infiltration are also present (hematoxylin and eosin, ×400).
		Claude	This kidney section also demonstrates diffuse glomerular abnormalities. The glomeruli show marked hypercellularity with an increase in mesangial matrix and cellularity. Capillary loops appear thickened and narrowed. Some glomeruli have a lobular appearance. Red blood cell casts are seen in some tubules. The interstitium shows edema and a patchy inflammatory infiltrate. These findings suggest an active proliferative glomerulonephritis, possibly post-infectious, IgA nephropathy, or lupus nephritis. Further workup with immunofluorescence and electron microscopy could help clarify the specific diagnosis.
83		Standard pathological description	microscopic polyangiitis. an active tubulointerstitial nephritis is frequently present with the glomerular lesions. There is a diffuse interstitial infiltrate of mononuclear cells and eosinophils associated with tubulitis (hematoxylin and eosin, ×200).
		Claude	The glomeruli in this specimen show similar hypercellularity and matrix expansion as the previous slide, but with more prominent lobular accentuation and segmental involvement. Some glomeruli have a crescent-like appearance at the periphery. The tubules contain proteinaceous casts. This pattern of injury is concerning for a crescentic glomerulonephritis, which can occur in ANCA-associated vasculitis, anti-GBM disease, or as a component of other glomerular diseases like IgA nephropathy or lupus nephritis. Prompt treatment is important to prevent rapid progression to end-stage kidney disease.
84	Hemoglobinuric acute renal failure	Standard pathological description	Hemoglobinuric acute renal failure. the hemoglobin casts have a fractured appearance (hematoxylin and eosin, ×400).
		Claude	This kidney biopsy demonstrates diffuse global glomerulosclerosis, with nearly all glomeruli showing advanced sclerosis and hyalinization. The architecture is distorted, with thickened glomerular basement membranes and obliteration of capillary loops. There is accompanying tubular atrophy and interstitial fibrosis, suggesting chronic injury. A few tubules contain proteinaceous casts. The blood vessels show thickening of the walls. Overall, this pattern is consistent with end-stage kidney disease, likely secondary to a chronic progressive glomerular disorder such as diabetic nephropathy, chronic glomerulonephritis, or hypertensive nephrosclerosis.
85		Standard pathological description	Hemoglobinuric acute renal failure. Higher-power image shows lysed reticulocytes forming an obstructing cast (hematoxylin and eosin, ×400).
		Claude	The glomeruli in this biopsy show diffuse and prominent hypercellularity, primarily within the mesangial regions and capillary loops. Many glomeruli have a lobular appearance with segmental accentuation. Capillary lumens are narrowed due to endothelial cell swelling and mesangial matrix expansion. A few glomeruli contain small cellular crescents at the periphery. The tubules show mild atrophy and contain red blood cell and proteinaceous casts. The interstitium is edematous with a mixed inflammatory infiltrate. These findings are consistent with an active proliferative and crescentic glomerulonephritis. Differential diagnosis includes IgA nephropathy, lupus nephritis, ANCA-associated glomerulonephritis, and post-infectious glomerulonephritis. Immunofluorescence and electron microscopy studies would help further characterize the nature of the immune deposits and ultrastructural features.
86		Standard pathological description	Thrombotic microangiopathy. Glomerulus demonstrates thickening of the capillary walls caused by endothelial cell swelling and accumulation of material between the endothelial cell and the basement membrane. Microthrombi are also present (hematoxylin and eosin, ×400).
		Claude	The slide shows kidney tissue with diffuse, nodular mesangial expansion and glomerular basement membrane thickening, resulting in a lobular appearance of the glomeruli. The capillary lumens appear narrowed. These features are consistent with diabetic nephropathy, specifically Kimmelstiel-Wilson lesions or nodular glomerulosclerosis. The surrounding tubules and interstitium also show some atrophic changes.
		Standard pathological description	Thrombotic microangiopathy. Glomerulus with focally bloodless capillaries with endothelial cell swelling and accumulation of material between the endothelial cell and the basement membrane. Microthrombi are also present (trichrome, ×400).

87	Thrombotic microangiopathy	Claude	This kidney tissue demonstrates membranoproliferative glomerulonephritis (MPGN) pattern. The glomeruli show marked mesangial proliferation with increased matrix, thickening of the capillary walls, and accentuation of lobular architecture. Capillary lumens are narrowed due to endocapillary proliferation and immune complex deposition. The "tram-track" or "double contour" appearance of the basement membrane is appreciable. Tubulointerstitial changes are also present.
88		Standard pathological description	Thrombotic microangiopathy. Glomerulus with focally bloodless capillaries caused by endothelial cell swelling and accumulation of material between the endothelial cell and the basement membrane. Microthrombi are also present (hematoxylin and eosin, ×400).
		Claude	The glomeruli in this slide exhibit a membranous nephropathy pattern. The capillary walls are uniformly thickened with a rigid appearance due to subepithelial immune complex deposition, best appreciated on silver stains. No significant hypercellularity or mesangial expansion is seen. Some capillary lumens remain patent. Tubular atrophy and interstitial fibrosis are present in the background.
89	subacute bacterial endocarditis–associated glomerulonephritis	Standard pathological description	subacute bacterial endocarditis–associated glomerulonephritis. a global proliferative pattern is present in this glomerulus, with scattered polymorphonuclear leukocytes (Jones silver stain, ×400).
		Claude	This slide shows a section of kidney tissue with several pathological changes indicative of membranous glomerulonephritis. The glomeruli appear enlarged and have thickened capillary walls with a "spike and dome" appearance, which is characteristic of this condition. There is also evidence of increased mesangial matrix and cellularity. The tubules and interstitium appear relatively unremarkable. Overall, the findings are consistent with a diagnosis of membranous glomerulonephritis, likely secondary to an underlying systemic disease or chronic infection.
90		Standard pathological description	subacute bacterial endocarditis–associated glomerulonephritis. The glomeruli in subacute bacterial endocarditis show hypercellularity, often with a membranoproliferative pattern, but in a focal and segmental distribution. There is often coexistence of segmental sclerosis. This glomerulus shows widespread glomerular basement membrane duplication and only rare polymorphonuclear leukocytes (Jones silver stain, ×400).
		Claude	This kidney pathology slide demonstrates changes consistent with membranoproliferative glomerulonephritis (MPGN). The glomeruli are enlarged and hypercellular, with a lobular appearance due to the increase in mesangial cells and matrix. The capillary walls are thickened and appear "split" or "tram-tracked," which is a hallmark feature of MPGN. There is also evidence of increased immune complex deposition along the capillary walls. The tubules and interstitium show mild atrophy and fibrosis. These findings support a diagnosis of MPGN, which may be primary or secondary to conditions such as systemic lupus erythematosus, hepatitis C, or complement disorders.
91		Standard pathological description	subacute bacterial endocarditis–associated glomerulonephritis. When the underlying valve lesion is not successfully treated, lesions may progress. in this case , the patient became septicemic and died weeks after renal biopsy. at autopsy, there was a diffuse proliferative glomerulonephritis with numerous polymorphonuclear leukocytes (Jones silver stain, ×400).
		Claude	This kidney pathology slide demonstrates changes consistent with membranoproliferative glomerulonephritis (MPGN). The glomeruli are enlarged and hypercellular, with a lobular appearance due to the increase in mesangial cells and matrix. The capillary walls are thickened and appear "split" or "tram-tracked," which is a hallmark feature of MPGN. There is also evidence of increased immune complex deposition along the capillary walls.
92	Scleroderma	Standard pathological description	Scleroderma. Fibrinoid necrosis of the arteriole is present, with fibrin thrombi occluding the lumen and extending to the hilus of the glomerulus. The glomerulus itself shows only ischemic change with corrugation of the glomerular basement membrane and retraction of the tuft (Jones silver stain, ×200).
		Claude	This slide shows kidney tissue with marked glomerular changes. The glomeruli appear enlarged and hypercellular, with increased mesangial matrix and numerous intracapillary and extracapillary proliferative lesions. Some glomeruli show crescent formation. The tubules are atrophic with interstitial inflammation and fibrosis. These findings are consistent with a proliferative and crescentic glomerulonephritis, possibly secondary to a systemic disease or vasculitis.
93		Standard pathological description	Scleroderma. in scleroderma crisis, there is fibrinoid necrosis of interlobular arteries and arterioles, with fibrin thrombi sometimes extending to glomeruli. There is ischemic tubular injury, and early interstitial edema and fibrosis (hematoxylin and eosin, ×100).
		Claude	This kidney pathology demonstrates similar changes to Image 1, with extensive crescent formation, marked glomerular hypercellularity, increased mesangial matrix, and focal areas of necrosis. The tubules show atrophy and there is interstitial inflammation. These features suggest a rapidly progressive crescentic glomerulonephritis. Differential diagnosis includes ANCA-associated vasculitis, anti-GBM disease, lupus nephritis, or IgA nephropathy.
94		Standard pathological description	Scleroderma. There is fibrinoid necrosis of the wall of this arteriole, with red blood cell fragments trapped within the wall and disruption of the internal elastic lamina (Jones silver stain, ×400).
		Claude	The biopsy here also reveals crescentic glomerulonephritis. Glomeruli are hypercellular with prominent cellular crescents surrounding the capillary loops. Some glomeruli appear partially sclerosed. The interstitium shows edema, inflammation and fibrosis. Tubular atrophy and thyroidization are present. The findings support a diagnosis of rapidly progressive glomerulonephritis, which requires further clinical correlation and serologic testing to elucidate the underlying etiology.
95		Standard pathological description	Scleroderma. even in glomeruli without demonstrable arteriolar injury or fibrin thrombi, there may be segmental corrugation and duplication of the glomerular basement membrane, related to subacute endothelial injury (Jones silver stain, ×400).
		Claude	Extensive glomerular involvement is seen with large cellular crescents compressing the capillary tufts. Focal fibrinoid necrosis is present. The interstitium contains a dense inflammatory infiltrate. Tubules are atrophic. These changes indicate severe crescentic glomerulonephritis consistent with ANCA-associated vasculitis or anti-GBM disease. Immunofluorescence and electron microscopy could help further subclassify the disease process.
96	kidney biopsy of healthy people	Standard pathological description	This paraffin-embedded 2- μm section illustrates a normal glomerulus with normal vascular pole with minimal periglomerular interstitial fibrosis and surrounding intact tubules. Mesangial cellularity and matrix are within normal limits (Jones silver stain, ×400).
		Claude	This slide shows a section of kidney tissue with numerous round to oval structures, which appear to be renal corpuscles (glomeruli). The glomeruli exhibit hypercellularity, with an increased number of cells within the tufts. There is also evidence of glomerular basement membrane thickening. The surrounding tubules appear dilated and contain eosinophilic material, possibly indicating proteinuria. These findings are consistent with a diffuse proliferative glomerulonephritis.
97		Standard pathological description	The normal glomerulus has thin, delicate glomerular basement membranes, three or fewer mesangial cell nuclei per mesangial area, and is surrounded by Bowman' s capsule. The adjacent tubules show a thin, delicate tubular basement membrane without lamellation or surrounding interstitial fibrosis. The vascular pole shows surrounding extraglomerular mesangial cells. The apparent mesangial cellularity of the glomerulus is highly dependent on the thickness of the section, and it is recommended that renal biopsies be cut at 2 μm thickness. This plastic embedded section is cut at 1 μm (Jones silver stain, ×400).
		Claude	The kidney tissue in this image demonstrates similar findings to Image 1, with prominent hypercellular glomeruli and thickened basement membranes. However, there appears to be more extensive tubular dilation and protein casts within the tubules. Additionally, some glomeruli show segmental sclerosis, suggesting a more advanced stage of glomerular injury. The overall pattern is suggestive of a proliferative glomerulonephritis with secondary tubular changes.
98		Standard pathological description	These glomeruli are from the same 28-week-gestation baby as shown in Fig. 2.6. They have more complex capillary branching pattern but maintain immature, plump glomerular visceral epithelial cells. In one glomerulus (on the right), the parietal epithelial cells are flattened and more mature in appearance (periodic acid Schiff, ×200).
		Claude	This slide reveals markedly enlarged glomeruli with significant hypercellularity, causing a lobular appearance. The capillary loops are obliterated due to the cellular proliferation. Tubules are dilated and contain proteinaceous material. There is also an increase in mesangial matrix. These features are characteristic of a membranoproliferative glomerulonephritis (MPGN).
99		Standard pathological description	During development, various stages of immature glomeruli may be found at different cortical levels within the kidney. The deep juxtamedullary glomeruli mature first. This immature glomerulus is from the midcortical level of a 28-week-gestation premature baby. There is prominent mesangium and very simple capillary branching with overlying plump, cuboidal glomerular visceral epithelial cells. The parietal epithelial cells lining Bowman' s capsule are also more cuboidal than in the mature state (periodic acid Schiff, ×400).
		Claude	The glomeruli in this section demonstrate a membranoproliferative pattern, similar to Image 3. The glomerular tufts are hypercellular and lobulated, with thickened capillary walls. Tubular dilation and protein casts are evident. In addition, there are areas of interstitial inflammation and fibrosis, indicating chronic damage. This slide suggests an MPGN with progressive tubulointerstitial changes.
100		Standard pathological description	The small but completely mature glomerulus of a normal term baby is illustrated, with complex capillary branching pattern and mature, pale-gray flattened podocytes overlying the capillary loops. The normal vascular pole is seen at the upper left. Normal proximal tubules with periodic acid Schiff–positive brush border with intervening peritubular capillaries are also illustrated. Although glomeruli do not increase in number with maturational growth, they increase in size. Normal glomerular diameter in children less than 5 years old in our biopsy practice is less than 95 μm. Individual laboratories must establish their own normal parameters because fixation and processing conditions may influence this parameter (periodic acid Schiff, ×100).

		Claude	This high-magnification view focuses on a single glomerulus with prominent hypercellularity and lobulation. The capillary lumens are narrowed due to endothelial and mesangial proliferation. The basement membranes appear thickened and split, creating a double-contour appearance (tram-track). These features are pathognomonic for MPGN, likely type 1.
